# Optimizing Organ Transplant Programs: A Comprehensive Review of Hospital Administrative Strategies and Clinical Outcomes

**DOI:** 10.7759/cureus.111028

**Published:** 2026-06-17

**Authors:** Manish Shrigiriwar, Amol Bhawane, Rahul Nayyar, Karthika Padmavathy, Veneel A Parikh, Sarada Prasanna Dash

**Affiliations:** 1 Department of Forensic Medicine, All India Institute of Medical Sciences, Nagpur, Nagpur, IND; 2 Department of Nephrology, All India Institute of Medical Sciences, Nagpur, Nagpur, IND; 3 Department of Hospital Administration, Mahatma Gandhi Medical College and Hospital, Jaipur, IND; 4 Department of Pathology, Sri Lalithambigai Medical College and Hospital, Dr. M.G.R. Educational and Research Institute, Chennai, IND; 5 Department of Community Medicine, Dr. N.D. Desai Faculty of Medical Science and Research, Dharmsinh Desai University, Nadiad, IND; 6 Department of General Surgery, Shri Jagannath Medical College and Hospital, Puri, IND

**Keywords:** clinical outcomes, donor allocation, governance structures, hospital administration, organ transplant management

## Abstract

Organ transplantation and hospital administration represent interdependent components of modern healthcare systems addressing end-stage organ failure. Growing procedural complexity, resource constraints, and persistent inequities highlight a gap in integrated understanding of how hospital-level administrative practices shape transplant performance beyond clinical interventions alone. This review aims to examine how organisational structures, leadership models, operational pathways, and digital infrastructure influence efficiency, equity, and clinical outcomes within transplant programs. A narrative review methodology was employed, drawing evidence from major biomedical and interdisciplinary databases covering publications from 2015 to 2025. The synthesis evaluated institutional governance, workforce management, information systems, quality frameworks, and patient-centred administrative practices across transplant settings. Findings indicate that coordinated governance, multidisciplinary leadership, standardised workflows, and robust health information systems are associated with improved donor utilisation, equitable access, outcome monitoring, and program sustainability. Administrative strategies supporting quality benchmarking and resource stewardship demonstrate relevance during periods of system stress and regulatory change. These observations emphasise aligning administrative design with clinical objectives to strengthen transplant delivery. The review highlights hospitals as stewards of complex transplant pathways, where organisational coherence influences patient and graft outcomes. Optimising transplant programs requires sustained integration of administrative innovation with clinical excellence to ensure resilient, equitable transplant systems globally.

## Introduction and background

Organ transplantation (OT) is among the most complex and resource-intensive fields of modern healthcare, requiring coordinated integration of clinical expertise, ethical governance, logistical coordination, and institutional management to achieve optimal patient and graft outcomes [1]. Advances in surgical practice, immunosuppressive therapy, perioperative care, and long-term follow-up have transformed transplantation into an established treatment option for selected patients with end-stage organ failure, with documented benefits for survival and quality of life [2]. Despite these advances, transplant systems continue to face a persistent imbalance between organ demand and organ availability, while hospitals must manage increasing operational complexity, regulatory obligations, and the growing use of digital and data-driven approaches in organ retrieval and transplantation [3].

For non-specialist readers, OT refers to the replacement of a failing organ with a functioning organ obtained from a living or deceased donor. In hospital-based practice, this pathway generally includes referral and evaluation of potential recipients, donor identification, organ retrieval, donor-recipient assessment, transplantation, perioperative care, immunosuppressive management, and long-term follow-up. Hospital administration supports this pathway by coordinating clinical departments, transplant teams, information systems, documentation, resource allocation, ethical oversight, and regulatory compliance. Although clinical outcomes after transplantation are extensively discussed in the literature, the administrative systems that support timely access, coordinated care, equitable allocation, and sustained follow-up require focused review.

The hospital serves as the cornerstone of the organ transplant system, where donor identification, recipient evaluation, organ retrieval, allocation-related coordination, transplantation, and aftercare are organised through interdependent clinical and administrative processes [4]. Hospital-level policies influence not only operational efficiency and compliance but also allocation practices, access pathways, outcome monitoring, and sustainability of transplant services [5]. Network design, procurement coordination, and allocation planning can affect transplant-system performance by shaping how organs, recipients, facilities, and transport logistics are matched across time-sensitive pathways [6]. As the volume of transplantations and the complexity of the patient population increase, hospital administration has moved beyond logistical support toward a broader coordinating role across multidisciplinary transplant care, donor systems, quality monitoring, and program-level accountability [7].

The existing body of knowledge is slowly laying more emphasis on the interaction of psychosocial, organisational, and system-level factors with transplant performance, including care coordination, information flow, process standardisation, and collaboration across departments [8]. Health information technology in transplant care has been reported to support data availability, communication, documentation, decision support, and continuity across pre-transplant, perioperative, and post-transplant stages [9]. These mechanisms operate through centralised access to donor and recipient clinical data, laboratory findings, immunological compatibility profiles, waitlist status, and logistical information required for organ allocation and retrieval. Digital platforms also strengthen communication among intensive care units, organ procurement teams, transplant coordinators, laboratories, surgical teams, and post-transplant follow-up services, thereby reducing duplication, delays, and fragmented decision-making. Process standardisation supports transplant performance by defining uniform steps for donor identification, candidate assessment, consent documentation, organ acceptance, perioperative preparation, and follow-up monitoring. Interdepartmental cooperation ensures that clinical, administrative, ethical, and logistical decisions are coordinated in real time rather than managed as isolated departmental activities. International recommendations on organ donation organisation architecture emphasise transparent governance, clearly defined responsibilities, coordination across donation and transplant systems, and accountability mechanisms as essential components of effective transplant programs [10]. These governance mechanisms support transparent prioritisation, documentation of allocation decisions, auditability of outcomes, and consistent application of ethical principles such as urgency, utility, equity, and fairness. Broader multi-stakeholder calls to action further underline that transplant performance depends on coordinated policy, professional, institutional, and public engagement rather than clinical expertise alone [11].

Policy and social factors also influence donation rates, consent processes, public trust, and access to transplantation [12]. Inequities in transplant access have been reported across socioeconomic, racial, geographic, and institutional contexts, indicating that disparities may arise not only from medical eligibility but also from referral pathways, evaluation processes, healthcare resources, and system-level barriers [13]. Addressing these inequities requires administrative attention to data monitoring, referral standardisation, patient navigation, community engagement, and alignment of institutional policies with broader equity-oriented transplant practices [14].

Clinical excellence and organisational performance are closely linked in transplantation, particularly during system stress, when hospital preparedness, resource mobilisation, infection control, and continuity planning affect service delivery [15]. Structured long-term management after kidney and liver transplantation requires coordinated monitoring of modifiable risks, graft function, complications, medication-related issues, and follow-up care within organised hospital systems [16]. Guideline-informed candidate evaluation and careful pre-transplant assessment support appropriate referral, listing, and follow-up, helping hospitals use scarce donor organs responsibly [17]. Fragmented workflows, inadequate coordination, and limited follow-up capacity can weaken transplant outcomes even when clinical expertise is available, particularly in recipients with complex cardiovascular and systemic risks [18].

Rising clinical complexity, including comorbidity burden, frailty, and variation in transplant-centre outcomes, reinforces the need for responsive administrative systems and structured performance monitoring [19]. The COVID-19 pandemic, specifically, demonstrated how disruptions in hospital preparedness, infection control, resource allocation, and continuity planning could affect solid OT services, underscoring the importance of organised leadership and flexible operational planning [20]. Best-practice recommendations in living kidney donation also emphasise education, access, donor care, and coordinated institutional processes, supporting the need for administrative-clinical integration across transplant pathways [21].

A comprehensive review of hospital administration and transplantation is timely because transplant outcomes are shaped by the interaction of clinical practice, organisational design, resource stewardship, digital infrastructure, governance, and equity-oriented access. By integrating evidence across these domains, this review seeks to clarify how hospital-level administrative strategies can support efficiency, accountability, sustainability, and patient-centred transplant care.

Objectives of the review

This review recapitulates existing evidence on the association between hospital administrative practices and the effectiveness, equity, and clinical outcomes of organ transplant programs. It examines the interaction between organisational structures, operational processes, and multidisciplinary coordination in shaping transplant performance outcomes. The review seeks to identify best-practice frameworks that can be applied to support healthcare institutions in optimising transplant delivery while achieving improved patient and graft outcomes.

Methodology

Review Design

This narrative review was designed to synthesise contemporary evidence on the relationship between hospital administrative strategies and clinical outcomes in organ transplant programs. A narrative review approach was selected because the topic spans heterogeneous domains, including hospital governance, transplant operations, workforce planning, health information systems, quality improvement, ethics, equity, and patient outcomes. This design allowed integration of evidence from empirical studies, systematic and scoping reviews, consensus statements, clinical guidelines, policy-oriented literature, and health systems research, which would not be easily captured through a narrowly defined meta-analytic framework. Although this was not designed as a systematic review or meta-analysis, the search, screening, eligibility criteria, and synthesis process were structured to improve transparency and reproducibility. Preferred Reporting Items for Systematic Reviews and Meta-Analyses (PRISMA) components were not applied because the manuscript was designed as a narrative review rather than a systematic review. Applying PRISMA standards would imply exhaustive systematic identification, formal study-selection reporting, risk-of-bias appraisal, and quantitative or structured evidence synthesis procedures that were not intended for this review design.

Search Strategy and Information Sources

A structured literature search was conducted across PubMed, Scopus, and Web of Science. These databases were selected because PubMed provides comprehensive coverage of biomedical, clinical, and transplantation literature; Scopus captures interdisciplinary health systems, management, and policy research; and Web of Science enables broader citation-based retrieval across clinical, administrative, and organisational science publications. The search covered publications from January 2015 to December 2025 to reflect contemporary transplant practice, including recent developments in digital infrastructure, organ allocation policy, quality benchmarking, and post-pandemic health system resilience.

Search terms were developed iteratively around three core concepts: OT, hospital administration, and clinical or operational outcomes. Boolean operators were used to combine keywords and related terms. Representative search strings included combinations such as: (“organ transplantation” OR “solid organ transplant” OR “kidney transplant” OR “liver transplant” OR “heart transplant”) AND (“hospital administration” OR “healthcare management” OR “governance” OR “leadership” OR “organizational structure” OR “program management”) AND (“clinical outcomes” OR “graft survival” OR “patient survival” OR “waitlist mortality” OR “readmission” OR “quality improvement” OR “equity” OR “access”). Additional search combinations included terms related to “donor identification,” “organ procurement,” “allocation policy,” “transplant coordinator,” “multidisciplinary team,” “health information systems,” “benchmarking,” “resource management,” and “patient-centred care.” Reference lists of relevant reviews, guidelines, and consensus documents were also screened manually to identify additional eligible sources. Because this was a narrative review, the search strategy was intended to identify conceptually and clinically relevant literature across administrative, organisational, policy, and transplant-care domains rather than to capture every eligible record through an exhaustive systematic review search.

Eligibility Criteria

Studies were included if they met the following criteria: publication between 2015 and 2025; focus on OT or transplant-related systems; discussion of hospital administration, governance, leadership, operational pathways, information systems, resource management, quality metrics, equity, or patient-centred administrative practices; and clear relevance to clinical, operational, ethical, access-related, or sustainability outcomes. Both adult and general transplant program literature were considered when findings had institutional or administrative relevance.

Exclusion criteria were applied to preserve the focus of the review. Articles were excluded if they focused exclusively on basic science, animal models, surgical technique without administrative relevance, pharmacologic management without organisational implications, or highly specialised clinical questions not linked to hospital-level processes. Publications were also excluded if they lacked sufficient methodological description, did not provide interpretable relevance to transplant program administration, or were not available in English. Duplicate records retrieved across databases were removed before screening.

Study Selection Process

The screening process was conducted in stages. First, titles and abstracts were reviewed to identify publications addressing hospital-level, organisational, administrative, technological, or policy-related factors relevant to transplant program performance. Second, potentially relevant full texts were assessed for their applicability to hospital-based transplant management and outcomes. Records identified through database searching and supplementary reference-list screening were reviewed sequentially, with duplicate removal occurring before title and abstract screening. Full-text articles were then assessed against the eligibility criteria. Because this was a narrative review, a PRISMA flow diagram was not prepared. Instead, the study-selection process is described narratively to clarify how relevant literature was identified, screened, and selected for thematic synthesis without presenting the work as a systematic review.

Data Extraction and Thematic Synthesis

Evidence synthesis was conducted thematically rather than statistically because of heterogeneity in study designs, transplant types, administrative models, health systems, and outcome measures. Extracted information was organised into major domains: evolution of hospital-based transplant programs; governance and organisational structures; leadership and multidisciplinary coordination; donor identification and procurement pathways; allocation policies; digital infrastructure; resource management and staffing; quality assurance and benchmarking; equity and patient-centred administration; and adaptive capacity and innovation. Within each domain, evidence was interpreted by identifying recurring administrative strategies, reported associations with operational or clinical outcomes, consistency across sources, and relevance to hospital-based transplant program optimisation. A formal study characteristics table was not developed because the included literature comprised diverse evidence types, including empirical studies, reviews, guidelines, consensus statements, policy-oriented sources, health systems research, and transplant-specific clinical evidence. These sources were used to support thematic narrative synthesis rather than comparative systematic tabulation.

Statistical pooling, meta-analysis, meta-regression, effect-size estimation, P-value reporting, and confidence-interval calculation were not performed because the included literature was heterogeneous in study design, transplant population, administrative focus, intervention type, and reported outcomes. The review therefore used narrative thematic synthesis to identify recurring administrative domains, evidence patterns, and clinically relevant associations rather than quantitative summary measures. Given this design, additional statistical review was not required.

Evidence Appraisal and Reproducibility Considerations

Formal risk-of-bias assessment was not performed because this manuscript was not designed as a systematic review and did not aim to compare individual study quality using a single appraisal framework. The included sources were heterogeneous in design and purpose, including guidelines, consensus statements, reviews, multicentre analyses, health information technology studies, policy-oriented literature, and clinical transplant evidence. Instead, evidence was appraised narratively according to relevance to hospital-level transplant administration, clarity of methods or recommendations, source type, recency, clinical or organisational applicability, and contribution to the thematic synthesis. Priority was given to peer-reviewed literature, clinical practice guidelines, consensus statements, systematic or scoping reviews, multicentre studies, and publications with clear institutional or health-system relevance. This approach supports transparency and interpretive reproducibility while remaining consistent with the narrative-review design.

Methodological Transparency

To enhance methodological transparency, priority was given to peer-reviewed studies, clinical practice guidelines, consensus statements, systematic or scoping reviews, multicenter analyses, and publications with clear institutional or health system implications. The synthesis did not aim to produce pooled effect estimates; rather, it sought to develop an integrative understanding of how administrative design and organisational practice influence transplant access, efficiency, quality, sustainability, and patient or graft outcomes. This approach is consistent with the purpose of a narrative review addressing a broad and multidisciplinary health systems topic. The reporting structure was strengthened by separating the review design, search strategy, eligibility criteria, study selection process, data extraction, evidence appraisal, and synthesis approach into distinct methodological subheadings.

## Review

Evolution of hospital-based organ transplant programs

The development of hospital-based transplant programs has progressed from procedure-centred surgical activity toward coordinated systems involving donor identification, recipient evaluation, perioperative care, post-transplant monitoring, ethical oversight, and institutional accountability. Structured donor-management protocols, health information technology, multidisciplinary coordination, immunosuppression management, and benchmarking processes have all contributed to this evolution by expanding transplant programs beyond surgery alone [4,13,14,19]. The COVID-19 pandemic further showed that transplant services depend on hospital preparedness, infection-control policies, resource mobilisation, and continuity planning during system disruption [15].

Transplant-system development also varies across national contexts. In India, hospital-based programs operate through a tiered structure involving the National Organ and Tissue Transplant Organization (NOTTO), Regional Organ and Tissue Transplant Organizations (ROTTOs), and State Organ and Tissue Transplant Organizations (SOTTOs), linking hospital activity with authorisation procedures, brain-stem death certification, organ-sharing coordination, and state-level implementation. In many high-income countries, deceased-donor registries, centralised allocation systems, digital integration, and organ procurement organisations are generally more mature. These differences influence donor identification, allocation, reporting, public-sector participation, and equity of access.

Structured governance and digitalisation represent important milestones in transplant-program development. Health information technology improves clinical data access, team communication, documentation, and follow-up continuity [4]. Allocation-policy literature shows that hospital practices translate broader allocation rules into practical decisions regarding candidate listing, organ acceptance, and outcome monitoring [5]. Economic pressures, resource constraints, and reimbursement considerations require hospitals to balance resource-intensive transplant care with quality, safety, and sustainability [2,21]. Recent work on organ donation and system efficiency also emphasises coordinated donation pathways, donor engagement, better organ utilisation, and integration between hospital processes and wider transplant networks [7]. Equity has become a central administrative concern because transplant access may vary according to socioeconomic, racial, geographic, and institutional factors [12]. Overall, hospital transplant programs are evolving toward integrated, data-informed, and accountable systems in which governance, clinical practice, quality monitoring, and equitable access are increasingly interdependent.

Organisational structures and governance models in transplant centres

The organisational structure of transplant centres determines how authority, accountability, clinical responsibility, and coordination are distributed across donation, procurement, allocation, transplantation, and follow-up activities [10]. Contemporary transplant programs increasingly depend on formal governance arrangements, defined roles, transparent reporting lines, and coordination mechanisms rather than informal leadership alone [10]. Clear governance structures support collaboration among donor-management teams, procurement services, clinical units, administrative leaders, and post-transplant follow-up services while helping hospitals translate allocation rules into practical decisions on candidate listing, organ acceptance, and outcome monitoring [5,10,11]. Effective communication systems are also necessary where responsibilities are distributed across multiple departments, because poor information flow can fragment transplant care despite the availability of clinical expertise and digital systems [4,9]. Formal transplant committees can support ethics review, clinical prioritisation, complex case evaluation, and alignment between institutional policy and clinical decision-making [10,17].

**Figure 1:**
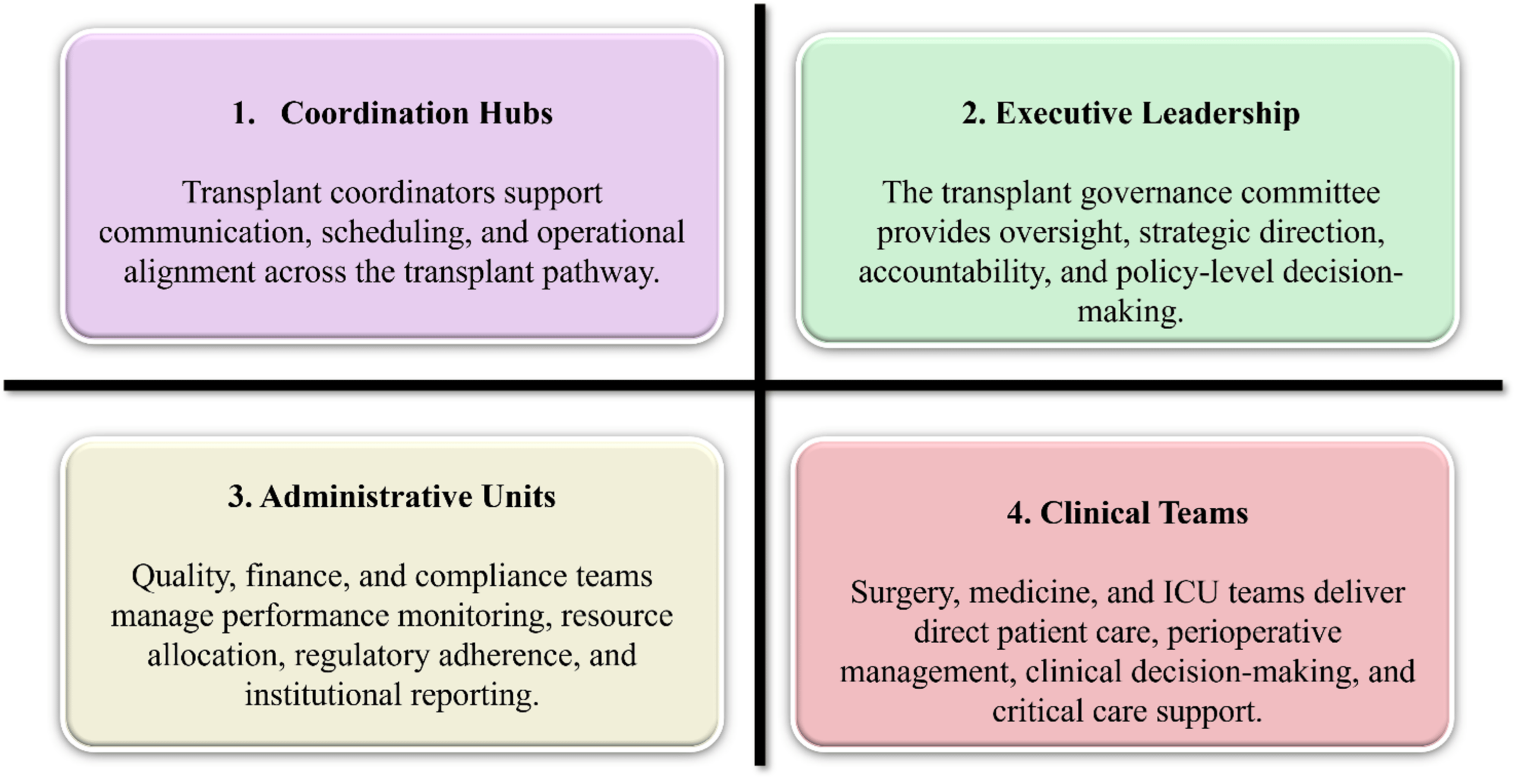
Structural Models of Hospital Transplant Program Governance Created by the authors using Microsoft PowerPoint

Network-based organisational planning improves coordination of organs, recipients, information, and resources across regional transplant systems [6]. Global transplant recommendations emphasise that transparent governance, public accountability, and stakeholder coordination are necessary to strengthen trust and support fair organ donation and allocation practices [11]. Administrative leadership further supports donor access, donor care, education, reporting, and institutional adherence to best-practice recommendations in transplant services [21]. At the system level, coordination between donor-management units, procurement pathways, and transplant services can improve donor participation, organ utilisation, benchmarking, equity monitoring, and overall system efficiency [7,12,19]. Effective and adaptive organisational structures therefore provide the administrative foundation for clinical quality, ethical accountability, coordinated service delivery, and sustainable transplant-program performance [7,10,11]. An example of transplant programs' institutional governance would be Figure 1.

Administrative leadership and multidisciplinary team coordination

Complex transplant environments require administrative leadership that can align clinical objectives with institutional responsibilities, resource availability, quality standards, and continuity of care [2]. Leadership supports cooperation among surgeons, physicians, transplant coordinators, nurses, pharmacists, laboratory teams, and ancillary services, while health information systems strengthen communication, documentation, data access, and follow-up continuity across the transplant pathway [2,4,9]. Multidisciplinary team models are especially important during candidate evaluation because guideline-informed assessment integrates medical suitability, risk evaluation, psychosocial considerations, and follow-up planning [17]. Leadership support also enables transplant coordinators to act as operational links between administrative structures and bedside care by supporting scheduling, documentation, patient education, interdepartmental communication, and follow-up continuity [4,21].

Regular multidisciplinary meetings support shared decision-making when medical urgency, ethical considerations, organ availability, allocation rules, and resource constraints must be balanced [10,11]. Team-based coordination supports adherence to clinical guidance, standardised monitoring, donor-management processes, and long-term management of modifiable post-transplant risks [14,16,21]. Organised follow-up systems are important for recipients with complex cardiovascular and systemic risks after transplantation [18]. Transparent leadership and clearly defined communication pathways reduce departmental silos and help hospitals translate allocation policies into candidate listing, organ acceptance, and monitoring decisions [5]. Overall, administrative leadership and multidisciplinary coordination provide the organisational basis for safer decision-making, continuity of care, ethical accountability, and resilient transplant-program performance [2,10,11].

Operational pathways in donor identification and organ procurement

Hospital operational pathways are central to timely donor identification, donor maintenance, procurement coordination, and subsequent organ availability [14]. Standardised donor-management protocols in intensive care settings support physiological stabilisation of potential donors and help preserve organ viability before retrieval [14]. Coordination among emergency departments, intensive care units, donor-management teams, laboratories, transplant coordinators, and surgical teams is administratively necessary for early recognition of eligible donors and timely escalation of transplant-related processes [4,9,14]. Well-established institutional processes also support ethical consent practices, documentation, communication with families, and alignment with broader organ-donation governance principles [10,11]. Dedicated transplant and donation coordination roles can improve communication, continuity, and system efficiency across donor identification, procurement, allocation, and follow-up pathways [4,7,21]. Procurement processes linked to regional or national sharing systems help improve allocation coordination, reduce avoidable delays, and support efficient use of donated organs [6,7,10].

Operational planning also requires early assessment of recipient-related clinical risks that may influence organ acceptance, perioperative planning, and post-transplant monitoring. In selected recipients, factors such as cancer history or exposure to immune checkpoint inhibitors require careful multidisciplinary review before transplantation [22]. Post-transplant lymphoproliferative disorder risk further illustrates the need for coordinated documentation, surveillance planning, and communication between transplant, pathology, oncology, and infectious disease services [23]. Pharmacogenomic and pharmacokinetic considerations, including CYP3A5-related tacrolimus variability, highlight the importance of linking procurement and transplantation workflows with medication-planning and therapeutic drug-monitoring systems [24]. Frailty assessment has also become relevant to candidate selection and perioperative risk planning, requiring structured communication between clinical teams and transplant-program administrators [25]. In living donor liver transplantation, technical and donor-related complexity reinforces the need for careful donor evaluation, operative planning, and coordinated institutional oversight [26]. Infection-risk planning is another administrative-clinical requirement, as cytomegalovirus prevention and monitoring depend on standardised protocols, laboratory coordination, and longitudinal follow-up systems [27]. Similarly, sarcopenia in liver transplant candidates requires multidisciplinary assessment because nutritional, functional, surgical, and follow-up considerations may influence perioperative risk and post-transplant recovery [28].

Operational resilience is also essential during system-level disruptions. The global impact of COVID-19 on solid OT demonstrated that procurement and transplant pathways require adaptable leadership, infection-control coordination, resource mobilisation, and continuity planning [29]. Consensus guidance on the use of hepatitis C viremic donors shows how new donor-utilisation strategies require coordinated consent, infectious disease input, recipient selection, and post-transplant monitoring [30]. Early COVID-19 experience among solid organ transplant recipients further emphasised the need for hospital-level coordination between transplant programs, infection-control teams, intensive care services, and follow-up systems [31]. Tacrolimus therapeutic drug-monitoring considerations also show that procurement and transplantation pathways must remain connected to post-transplant medication management, laboratory systems, and long-term safety monitoring [32]. Overall, donor identification and organ procurement depend on close integration between hospital administration, clinical teams, digital systems, ethical governance, recipient-risk assessment, and regional or national transplant networks [4,6,7,10,14]. Table 1 summarises the key administrative processes influencing donor identification and organ procurement efficiency.

**Table 1:** Administrative Processes Influencing Donor Identification and Organ Procurement Efficiency ICU: Intensive Care Unit; ED: Emergency Department

Administrative Domain	Key Hospital-Level Processes	Operational Impact	Clinical Implications	References
Donor Identification	Early recognition protocols in ICU and emergency settings	Timelier identification of potential donors	Improved organ availability and reduced missed donation opportunities	[14]
Donor Management	Standardised physiological stabilisation pathways for potential donors	Improved donor maintenance and organ viability	Better preservation of transplantable organs before retrieval	[14]
Consent Coordination	Structured family communication, ethical oversight, and legal documentation	More consistent authorisation processes	Improved transparency, ethical consistency, and public trust	[10,11]
Interdepartmental Coordination	Integration of ICU, procurement, laboratory, surgical, and transplant teams	Improved communication and reduced workflow fragmentation	More coordinated procurement, allocation, and perioperative planning	[4,9,14]
Network Integration	Alignment with regional and national organ-sharing systems	Improved organ movement, matching, and allocation coordination	Increased system efficiency and more effective organ utilisation	[6,7,10]

Hospital policies and allocation practices affecting clinical outcomes

Hospital-level policies translate national or regional allocation frameworks into practical decisions on candidate listing, organ acceptance, prioritisation, documentation, and outcome monitoring [5]. These policies help balance medical urgency, expected transplant benefit, ethical considerations, logistical feasibility, and institutional capacity while reducing unwarranted variation in listing, organ acceptance, and follow-up expectations [5,6,10,17]. Hospitals therefore act as operational intermediaries between broader allocation systems and individualised clinical judgment, especially when evaluating marginal organs, high-risk recipients, and specialised indications [10,17,33]. Pregnancy after kidney transplantation and immune checkpoint inhibitor exposure in transplant recipients illustrate situations where institutional policies must support structured counselling, multidisciplinary review, risk assessment, and follow-up planning [34,35].

Institutional compliance mechanisms support consistent application of allocation criteria, transparent documentation, and public trust in transplant systems [11]. Equity-focused policies are also necessary because inequities in referral, evaluation, and waitlisting have been reported across socioeconomic, racial, geographic, and institutional contexts [12]. Major system disruptions, including COVID-19, further demonstrated the need for flexible hospital policies that preserve ethical allocation, infection-control safety, and continuity of transplant services during changing clinical conditions [36]. Policies aligned with broader allocation frameworks can therefore make regulatory principles more consistent, transparent, equitable, and clinically actionable within hospital-based transplant programs [5,7,10,11].

Integration of health information systems and digital infrastructure

Health information systems have become central to transplant care because they support data access, communication, documentation, and continuity across pre-transplant evaluation, perioperative care, post-transplant monitoring, and long-term follow-up [4,9]. Electronic health records and transplant-specific digital platforms help multidisciplinary teams coordinate donor and recipient information, laboratory findings, imaging results, medication records, immunological data, and follow-up documentation [4]. These systems also strengthen administrative oversight by improving traceability, standardised documentation, and communication among transplant coordinators, clinical departments, laboratories, procurement services, and follow-up teams [4,9]. Integrated data infrastructure is especially important for immunosuppression management, quality-of-life follow-up, graft-injury surveillance, infection monitoring, medication-level tracking, and prediction of graft-survival risk [37-43].

**Figure 2:**
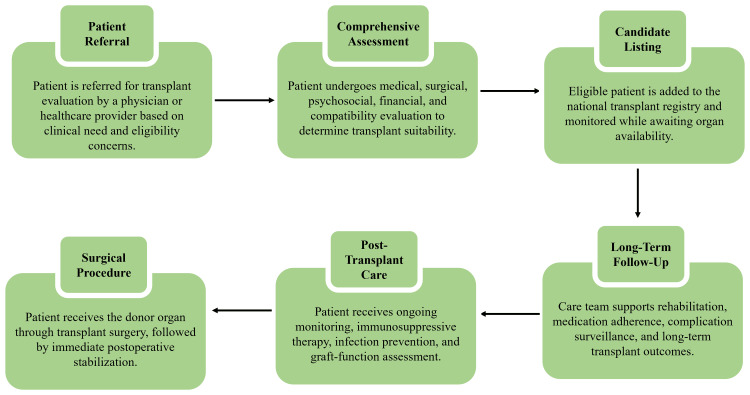
Digital Information Flow Across the Transplant Care Continuum Created by the authors using Microsoft PowerPoint

Digital infrastructure also supports procurement and allocation workflows by improving communication between donor hospitals, transplant centres, laboratories, and allocation networks in time-sensitive decisions [4,6,9,14]. Overall, digital infrastructure strengthens hospital administration by enabling data-driven monitoring, regulatory documentation, quality assurance, patient follow-up, and coordinated clinical decision-making across contemporary transplant programs [4,9,19,43]. Figure 2 is a representation of data that includes stages of transplant care.

Resource management, staffing, and financial sustainability

Transplant programs require effective resource management because they involve high-cost clinical care, specialised staff, intensive monitoring, operating-room access, intensive care capacity, laboratory support, and long-term follow-up [2,21]. Hospital administration must balance these resource demands with patient safety, equitable access, regulatory compliance, and institutional sustainability [2,10,21]. Transplant-specific coordinators, nursing teams, pharmacists, laboratory personnel, and follow-up services support continuity of care across donor identification, surgery, immunosuppression management, complication monitoring, and outpatient review [4,16,21]. Financial planning in transplant services includes budgeting, reimbursement awareness, cost monitoring, infrastructure allocation, and evaluation of downstream costs such as readmissions and prolonged hospitalisation [5,44]. Benchmarking literature shows that transplant outcomes vary across centres, suggesting that differences in institutional resources, perioperative systems, case selection, and quality monitoring may influence performance [19]. Administrative oversight also supports appropriate allocation of operating-room time, intensive care resources, procurement logistics, laboratory capacity, and outpatient follow-up services [10,14].

Economic and operational pressures are particularly relevant because readmissions after liver transplantation create substantial variation in centre-level burden and healthcare costs [44]. Hospitals that invest in workforce training, role clarity, coordinated donor-care processes, and patient education may improve program stability and continuity of transplant services [2,21]. Interdisciplinary staffing models support coordination among procurement, surgical, medical, pharmacy, laboratory, and follow-up teams, thereby improving program functionality across the transplant pathway [4,7,16]. Resource planning also needs to account for advanced transplant technologies; for example, portable normothermic machine perfusion illustrates how innovation may improve preservation strategies while requiring institutional investment, staff training, and operational planning [45]. Quality-linked monitoring, outcome benchmarking, and cost surveillance help administrators assess whether resource-intensive services are producing acceptable clinical value and sustainable program performance [19,21,44]. Resource-conscious management is also relevant to equity because limited institutional capacity can affect referral, evaluation, waitlisting, and timely transplantation for underserved patient groups [12]. Integrated workforce and financial planning therefore strengthen the ability of transplant programs to maintain quality, continuity, access, and sustainability in resource-intensive care environments [2,7,10,21,44]. As indicated in Table 2, the structure of the workforce and fiscal planning affect the stability and performance of the transplant services.

**Table 2:** Resource Allocation and Staffing Strategies in Hospital Transplant Programs ICU: Intensive Care Unit; OR: Operating Room

Resource Dimension	Administrative Strategy	Operational Outcome	Financial/Clinical Benefit	References
Workforce Planning	Dedicated transplant coordinators, nursing teams, and follow-up personnel	Improved care coordination and continuity	More consistent patient navigation, documentation, and follow-up	[4,21]
Staffing and Workload Alignment	Matching transplant workload with clinical, laboratory, pharmacy, ICU, and outpatient capacity	Reduced workflow fragmentation and improved service continuity	More reliable perioperative care and long-term monitoring	[2,16,19]
Financial Oversight	Integrated budgeting, cost monitoring, and readmission surveillance	Improved awareness of resource use and cost burden	Better sustainability planning for resource-intensive transplant services	[44]
Infrastructure Utilisation	Optimised operating-room, ICU, procurement, laboratory, and outpatient scheduling	Improved use of high-cost hospital infrastructure	More coordinated transplant delivery and reduced avoidable delays	[10,14]
Technology and Outcome-Linked Resource Planning	Investment planning for quality monitoring, benchmarking, and advanced preservation technologies	Improved performance tracking and operational preparedness	Support for clinical value, organ preservation, and long-term program sustainability	[19,21,45]

Quality assurance, performance metrics, and benchmarking

Transplant programs require quality assurance frameworks that monitor safety, effectiveness, consistency, and outcomes across the transplant pathway [19]. Performance measures such as graft survival, patient survival, complications, readmissions, infection-related outcomes, and long-term functional status help hospitals evaluate whether transplant services are achieving acceptable clinical results [16,19,44]. Benchmarking allows transplant centres to compare outcomes, identify performance gaps, and define best achievable results, as demonstrated in multicentre liver transplantation analyses [19]. Standardised indicators also support transparent reporting, regulatory accountability, and structured governance in organ donation and transplantation systems [10,11]. Hospitals that adopt quality committees, audit systems, adverse-event reporting, and feedback mechanisms may be better positioned to respond to safety events, process deviations, and opportunities for quality improvement [10,19,21]. Monitoring across the care continuum can support earlier recognition of immunosuppression-related complications, infection risks, readmissions, medication-level variability, and graft-injury signals [13,40-42]. Digital quality dashboards and health information systems can further support real-time monitoring, documentation, communication, and longitudinal tracking of transplant outcomes [4,9].

**Figure 3:**
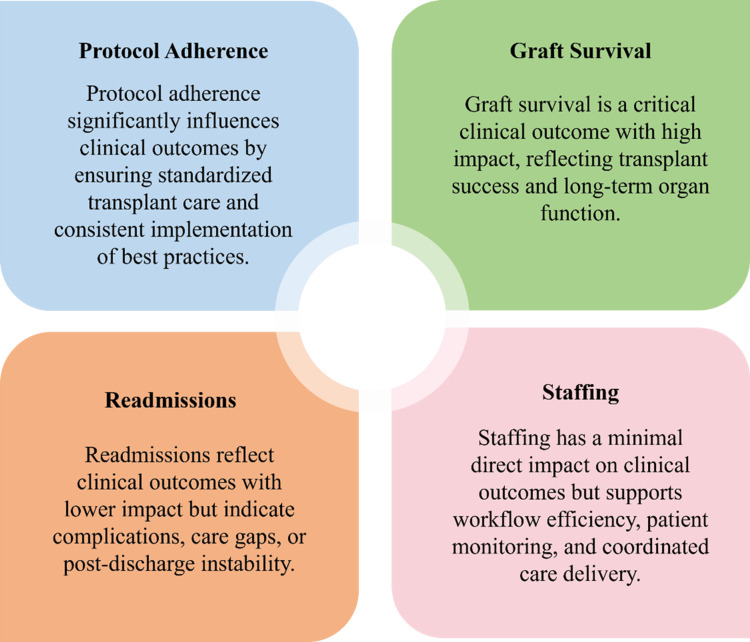
Relationship Between Administrative Performance Metrics and Clinical Outcomes Created by the authors using Microsoft PowerPoint

Patient-reported outcomes and quality-of-life measures add an important patient-centred dimension to transplant quality assessment, particularly in long-term follow-up after kidney transplantation [39]. Continuous feedback between administrative leadership and clinical teams can strengthen adherence to allocation policies, clinical guidelines, documentation standards, and evidence-informed transplant practices [5,16,17]. Readmission monitoring is also important because post-transplant readmissions create measurable variation in economic burden across centres and can signal opportunities for quality improvement [44]. Participation in national or regional transplant registries and transparent reporting systems can support shared learning, accountability, and comparison of outcomes across programs [10,11]. Risk-adjusted prediction models and decision-support approaches may improve benchmarking accuracy by accounting for recipient characteristics, case complexity, and expected graft-survival probability [43]. Quality assurance and benchmarking therefore function as core administrative tools for sustaining clinical performance, ethical accountability, resource stewardship, and continuous improvement in hospital-based transplant programs [7,10,19,21]. The links between the administrative indicators and clinical outcomes are presented in Figure 3.

Equity, access, and patient-centred administrative practices

Equitable access to OT is increasingly recognised as a core quality concern because referral, evaluation, waitlisting, and transplantation may vary across socioeconomic, racial, geographic, and institutional contexts [12]. Hospital-level referral and evaluation pathways determine which patients are assessed for transplant candidacy and how consistently they progress through eligibility review, risk assessment, and waitlist registration [17]. Scoping-review evidence indicates that access disparities may reflect system-level and institutional barriers, not only differences in medical eligibility [12]. Standardised intake, evaluation, documentation, and listing procedures can reduce unwarranted variation and improve transparency in access to transplant services [5,17]. Administrative engagement in education, counselling, and access support is also important in living donation and transplant pathways, where informed decision-making and donor-recipient support require organised institutional processes [21]. Patient-centred administrative models rely on communication, navigation, scheduling support, education, and coordination across the transplant pathway [2,4]. Specialised transplant coordinators can reduce procedural complexity by helping patients navigate appointments, documentation, communication with teams, and follow-up requirements [4].

Equity-oriented hospital policies may support more consistent referral, evaluation, and waitlisting practices while strengthening public trust in transplant systems [11,12]. Psychosocial evaluation and support are also relevant because mental health, coping capacity, adherence, family support, and social circumstances can influence transplant preparedness and long-term outcomes [8]. Patient-centred assessment should also include quality-of-life outcomes because long-term recovery after kidney transplantation involves functional, psychological, and social dimensions beyond graft survival alone [39]. Administratively monitored demographic, socioeconomic, referral, waitlisting, and outcome data can help institutions identify access gaps and design targeted corrective actions [12,21]. Vaccination and infection-prevention planning in chronic kidney disease and kidney transplantation further illustrate how patient-centred administration must coordinate education, preventive care, and follow-up for vulnerable transplant populations [46]. Hospital-based equity and patient-centred practices therefore support social accountability, transparent access, informed participation, continuity of care, and long-term transplant outcomes [2,4,11,12,17,21]. Table 3 highlights management plans that support equal access and personalised services in the transplant care pathways.

**Table 3:** Administrative Interventions Targeting Equity and Patient-Centred Care

Equity Focus Area	Administrative Intervention	Access-Related Outcome	Patient-Centered Impact	References
Referral Pathways	Standardised intake, evaluation, and waitlisting protocols	Reduced unwarranted variation in referral and listing decisions	More transparent and consistent access to transplant evaluation	[12,17]
Patient Education and Access Support	Structured counselling, education, and donor-recipient support processes	Improved informed participation in transplant pathways	Better understanding of evaluation, donation, and follow-up requirements	[21]
Care Navigation	Dedicated transplant coordinators and communication support	Reduced procedural burden and workflow fragmentation	Improved patient navigation, documentation, and follow-up continuity	[4]
Psychosocial Support	Integrated psychosocial assessment and support workflows	Earlier identification of adherence, coping, and social-support needs	More holistic preparation and long-term support after transplantation	[8]
Equity Monitoring	Demographic, socioeconomic, referral, waitlisting, and outcome-data tracking	Identification of institutional access gaps and disparities	Targeted corrective actions to improve fairness and accountability	[12,21]

Adaptive capacity and innovation in hospital transplant program management

Adaptive capacity in hospital transplant programs refers to the ability of administrative systems to adjust governance, workflows, staffing, technology, and clinical coordination in response to changing donor availability, recipient complexity, regulatory requirements, and system-level pressures [47]. Innovation-oriented leadership supports the introduction of new technologies, care models, quality-improvement processes, and performance-feedback systems into routine transplant operations [2,4,19,21]. Data-driven tools can assist with risk stratification, medication monitoring, follow-up planning, quality-of-life assessment, and graft-survival prediction when integrated with clinical judgment and governance oversight [24,39,43]. Administrative flexibility is also required when programs adopt complex donor-utilisation or high-risk recipient strategies, including hepatitis C viremic donor transplantation, immune checkpoint inhibitor exposure, and complex liver-transplant indications [30,35,48].

Adaptive programs also require coordinated infection-prevention, laboratory, pharmacy, procurement, surgical, and follow-up pathways, particularly during system disruptions and evolving infectious risks [4,16,27,49]. Artificial-intelligence-based analytical approaches illustrate the potential role of computational tools in transplant data analysis and decision support, although validation and governance oversight remain necessary before routine administrative use [3,50]. Overall, adaptive and innovative management systems support long-term effectiveness, operational sustainability, clinical safety, and resilience by integrating governance, digital infrastructure, quality monitoring, and multidisciplinary coordination [2,4,7,10,19,21].

Limitations and future recommendations

Several limitations should be considered when interpreting the findings of this review. First, the narrative design was intended for synthesis and interpretation rather than quantitative pooling; therefore, the findings may be influenced by author interpretation despite the use of structured search, screening, and eligibility procedures. Second, heterogeneity across transplant types, hospital settings, administrative models, health systems, and outcome measures limited direct comparison across studies and prevented causal inference. Third, methodological variation among the included sources, including differences in study design, data quality, reporting detail, and outcome definitions, may affect the consistency of interpretation. Finally, much of the available literature reflects experience from high-income healthcare systems with relatively developed transplant infrastructure, which may limit transferability to low- and middle-income settings.

Future research should prioritise rigorous multicentre studies that directly evaluate administrative interventions and their long-term effects on clinical, operational, financial, and equity-related outcomes. Greater standardisation of terminology, performance indicators, reporting frameworks, and outcome measures would improve comparability across transplant programs and strengthen future evidence synthesis. Further research is also needed in geographically diverse and resource-limited health systems to improve generalisability and identify context-specific administrative strategies. Mixed-methods designs combining quantitative outcome assessment with qualitative organisational analysis may provide deeper insight into how governance, leadership, staffing, digital infrastructure, and patient-centred practices influence transplant-program performance. Closer collaboration among clinicians, administrators, data scientists, health-system leaders, and policy makers will be important for translating evidence into scalable, adaptive strategies that improve transplant outcomes and long-term program sustainability in changing regulatory and technological environments.

## Conclusions

Organ transplant programs function at the interface of advanced clinical practice and complex hospital administration, making effective alignment between medical expertise and organisational strategy essential. This review demonstrates that governance structures, leadership engagement, operational pathways, digital infrastructure, resource planning, quality assurance, and equity-oriented practices all influence transplant efficiency, access, sustainability, and clinical outcomes. Well-designed administrative frameworks support multidisciplinary coordination, ethical allocation, donor identification, transplantation, and long-term follow-up. They also strengthen data-informed decision-making, workforce stability, benchmarking, patient navigation, and adaptive capacity in resource-intensive transplant environments. Overall, clinical excellence in transplantation is inseparable from institutional design, managerial coherence, and administrative resilience. Hospitals therefore serve as key stewards of scarce donor organs and complex care pathways, and sustained alignment between administrative processes and clinical objectives is necessary to support effective, equitable, and resilient transplant systems across diverse healthcare settings.
